# Potential of Wake-Up Radio-Based MAC Protocols for Implantable Body Sensor Networks (IBSN)—A Survey

**DOI:** 10.3390/s16122012

**Published:** 2016-11-29

**Authors:** Vignesh Raja Karuppiah Ramachandran, Eyuel D. Ayele, Nirvana Meratnia, Paul J. M. Havinga

**Affiliations:** Pervasive Systems Research Group, University of Twente, Enschede 7522NB, The Netherlands; e.d.ayele@utwente.nl (E.D.A.); n.meratnia@utwente.nl (N.M.); p.j.m.havinga@utwente.nl (P.J.M.H.)

**Keywords:** wake-up radio, Implantable Body Sensor Networks, Medium Access Control, closed loop medical devices

## Abstract

With the advent of nano-technology, medical sensors and devices are becoming highly miniaturized. Consequently, the number of sensors and medical devices being implanted to accurately monitor and diagnose a disease is increasing. By measuring the symptoms and controlling a medical device as close as possible to the source, these implantable devices are able to save lives. A wireless link between medical sensors and implantable medical devices is essential in the case of closed-loop medical devices, in which symptoms of the diseases are monitored by sensors that are not placed in close proximity of the therapeutic device. Medium Access Control (MAC) is crucial to make it possible for several medical devices to communicate using a shared wireless medium in such a way that minimum delay, maximum throughput, and increased network life-time are guaranteed. To guarantee this Quality of Service (QoS), the MAC protocols control the main sources of limited resource wastage, namely the idle-listening, packet collisions, over-hearing, and packet loss. Traditional MAC protocols designed for body sensor networks are not directly applicable to Implantable Body Sensor Networks (IBSN) because of the dynamic nature of the radio channel within the human body and the strict QoS requirements of IBSN applications. Although numerous MAC protocols are available in the literature, the majority of them are designed for Body Sensor Network (BSN) and Wireless Sensor Network (WSN). To the best of our knowledge, there is so far no research paper that explores the impact of these MAC protocols specifically for IBSN. MAC protocols designed for implantable devices are still in their infancy and one of their most challenging objectives is to be ultra-low-power. One of the technological solutions to achieve this objective so is to integrate the concept of Wake-up radio (WuR) into the MAC design. In this survey, we present a taxonomy of MAC protocols based on their use of WuR technology and identify their bottlenecks to be used in IBSN applications. Furthermore, we present a number of open research challenges and requirements for designing an energy-efficient and reliable wireless communication protocol for IBSN.

## 1. Introduction

The human population is growing at an alarming rate. This rapidly growing population has resulted in new health problems due to various factors such as demographic aging, rapid urbanization, and the spread of unhealthy lifestyles. According to the World Health Organization (WHO), non-communicable diseases, such as cardiovascular disease, cancer, diabetes and chronic lung diseases, have overtaken infectious diseases as the world’s leading cause of mortality [1]. Innovations in the health-care industry are continuously emerging to monitor, treat and reduce the death and disability caused by such non-communicable diseases. With the advent of nano-technology, medical sensors and devices are becoming highly miniaturized [2]. The size of the sensor nodes is also being reduced, which allows them to be implanted inside the body using minimal invasive surgery. The advantage of being closer to the cause of a disease will increase the understanding of the pathological symptoms of the disease [3].

In general, the advantages of miniaturized implantable sensors over external sensors include (i) their accurate diagnosis of a symptom; (ii) being unobtrusive compared to the body-worn sensors; and (iii) having minimal impact on the quality-of-life of a patient. While some implantable medical devices, such as pace-makers, are known already for more than five decades, many new implantable devices, such as drug-delivery devices, neural- and deep-brain simulators have only been introduced in the last two decades. It is important to note that the traditional implantable devices had neither complex computational operations nor the demanding RF-communication capabilities. These additional functionalities of the advanced medical implants can cause bio-compatibility issues such as tissue burns due to thermal overloading [4]. Also, usual bio-compatibility problems, such as fibrous encapsulation, and calcification of implants can have a negative impact on the performance of advanced implantable devices [4]. Similarly, continuous exposure of tissues to radio waves can cause surface heating of the tissues around the implant [5]. Although there are different biochemical ways to overcome the bio-compatibility problems [4], it is crucial to handle the problems with engineering solutions. For example, multi-sensor arrays can be used to reduce the measurement errors caused by bio-fouling of sensor nodes [6]. These methods not only improve the accuracy of the measurements, but also eliminate the need of complex processing of the data; doing so will prevent over-heating of the sensor nodes.

While, traditionally, many implantable medical devices have operated in isolation, recently some attempts have been made to connect them and form a network of implantable medical devices and sensors, the so-called IBSN. An IBSN is very different from the network of body-worn sensor nodes because of its special characteristics and requirements. Table 1 shows the typical differences between IBSN and BSN.

### 1.1. Closed-Loop Medical Devices

Recently, the so-called closed-loop medical devices [7] have been highlighted [8]. The closed-loop medical devices can deliver a therapy autonomously with the feedback from bio-sensors monitoring the physiological signals. A pace-maker is a well-known closed-loop medical device that is capable of fibrillating or defibrillating the heart when the heart beat of a patient becomes slower or faster, respectively. In this case, the pace-maker has a heart-rate sensor attached to the heart which is hardwired together with the pacing leads.

Modern closed-loop medical devices are not only capable of providing therapies for heart diseases but also for more complex diseases. For example, in the case of Parkinson’s Disease, Deep Brain Stimulator (DBS) can autonomously adjust the stimulation parameters to stabilize the tremors in real-time. This is achieved by continuously sensing the tremors in a closed-loop fashion. The symptoms of Parkinson’s patients are not present in close proximity to the therapeutic location. In most cases, the sensor is placed in the limbs of the patient to monitor the tremor. In such a case, a wired connection is not possible between the DBS and sensor nodes. Other closed-loop medical devices, such as ‘neural bypass’ which connects the brain directly to the muscles, by-passing the spinal cord in case of the paralytic patients [9], artificial limbs, drug delivery devices, nerve stimulators have also recently been explored [10,11,12].

Those implantable medical devices that monitor symptoms of the diseases using a wired sensor in close proximity of the therapeutic device do not require a wireless communication, while a wireless connection is inevitable for medical devices that monitor symptoms of the diseases that are not present in close proximity of the therapeutic location.

Due to the fact that the number of sensors required to accurately prognose a disease is increasing, currently more than one medical device is used to deliver a therapy. An energy-efficient and reliable wireless communication mechanism is mandatory for the real-time flow of data between these heterogeneous medical sensor nodes. The closed-loop medical systems inherit the properties of a traditional closed-loop control system. The prime characteristics of such systems are presented in Table 2.

### 1.2. Challenges of MAC Protocol for IBSN

One of the challenging objectives of wireless communication protocols for IBSN is to be ultra-low-power. Wireless communication in IBSN suffers from three main problems, which not only affect the power consumption of sensor nodes but also reliability and quality-of-service of wireless communication inside the body. These main problems are: (i) idle listening which occurs when the node listens for data-packets while no data-packets are being sent; (ii) overhearing, which occurs when the node is listening to data-packets which are not destined for it; and (iii) packet collision, which occurs when two nodes compete to transfer at the same time through the same channel. In general, MAC protocols are designed to reduce these problems by regulating the access to the wireless medium. In the case of IBSN, additional challenges are faced, for example signal attenuation due to the dynamic movements of the human body, very high attenuation of signals due to the conductive nature of the human tissues, and heterogeneous requirements of different devices that are present in the network. Apart from the traditional carrier sensing and time division methods, new technologies that enhance the MAC protocol design, such as WuR, are being currently researched.

### 1.3. Impact of WuR in the Design of MAC Protocols

**Figure 1:**
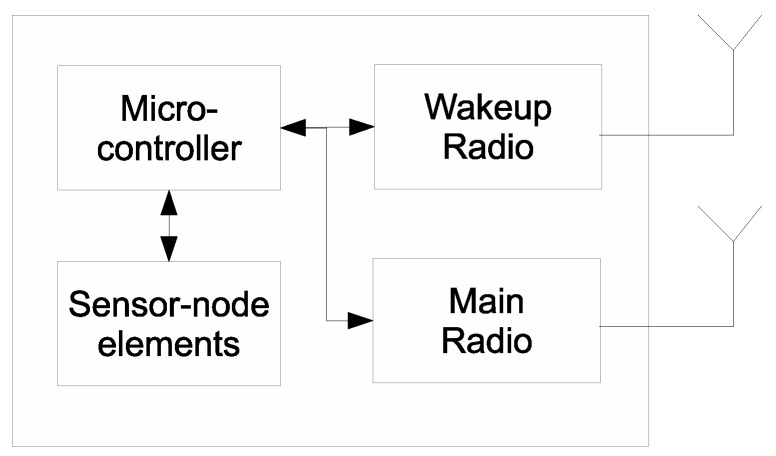
Common architecture of a sensor node with WuR.

In the last decade, the concept of ultra-low-power WuR [13] was introduced. Schematic architecture of the sensor node with WuR is shown in Figure 1. WuR operates together with the main radio with much lower power consumption than the main radio. The WuR reduces the energy consumption of wireless communication by switching the main radio to deep-sleep mode when no data is transmitted or received. It is operated with different duty-cycles and yields a good power budget for the sensor nodes. The WuR can either operate in the same band of the main radio or in a different band. Different innovative WuR designs already exist, which operate in the power range of nanowatts [14]. It has been shown that WuR reduces the overall power consumption of wireless communication in sensor nodes, provided that the duty-cycle of main radio is carefully selected and synchronized with the duty-cycle of the WuR [15].

The main features of WuR to make it as a suitable technology for MAC protocols of IBSN are its low power consumption, reliable performance in short-range networks, ability to operate out-of-band with main radio, and not requiring complex hardware [16]. By turning the main radio on when it is really needed, WuR limits the power consumed by the main radio for idle listening along with the over-hearing problems preventing data collision from occurring. The data communication is then initiated and completed using the main radio, reducing the total amount of time that the main radio is turned on. The WuR of the transmitter broadcasts a node-id encoded wake-up signal, which is acknowledged by the WuR of the destined node, indicating that the main radio of the receiver node is actively listening. The main radio of the transmitter is turned on only when the acknowledgment is received for the wake-up signal, hence reducing energy consumption and increasing reliability.

### 1.4. Contributions

In this survey, we primarily focus on the MAC protocols that are applicable to IBSN. Although numerous MAC protocols are available in the literature, the majority of them are designed for BSN and WSN. To the best of our knowledge, there is so far no research paper that explores the impact of these MAC protocols specifically for IBSN. In this context, the main contributions of this paper are:

- Identifying requirements of MAC protocols for IBSN,
- Providing a taxonomy for the existing MAC protocols based on the WuR technology,
- Identifying the research challenges in the design of MAC protocols for IBSN.

The rest of the paper is organized as follows. We first explain the characteristics of IBSN in Section 2, with respect to the general strategies of IBSN and elucidate its difference from conventional BSN. In Section 3, we present the traditional medium access mechanisms and their working principle concerning the IBSN. In Section 4, we categorize the existing MAC mechanisms based on their use of WuR and present a taxonomy followed by a comparison between the access mechanism in the classified order. An evaluation of MAC protocols based on the requirements of IBSN is presented in Section 5. Eventually, we present the open research challenges in designing MAC protocols for IBSN in Section 6, followed by a concluding remark from our study which is included in Section 7.

## 2. Architectural Framework of IBSN and Its Components

The building components of the IBSN, such as the sensors for measuring physiological data, the medical devices for delivering medical therapies, the implantable radio, and the microprocessor are commercially available. Functionality of these components is trivial, however the environment and the requirements of IBSN make the functionality of these devices non-trivial. For a closed-loop operation, it is important that the components of the IBSN are integrated in an optimized architecture to ensure reliability, energy-efficiency, and QoS. The architectural framework of IBSN will define:

- Sensing strategies
- Actuation strategies
- Power scavenging and energy-efficiency strategies
- Data handling strategies
- Communication strategies

**Figure 2:**
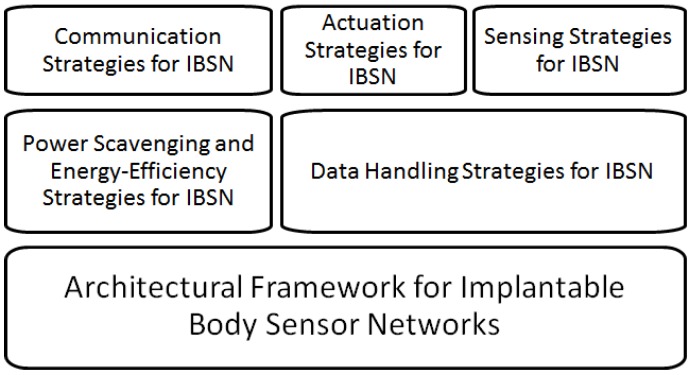
Common architecture of IBSN sensor nodes.

The common architecture of the closed-loop operation is shown in Figure 2. In the following sections, we briefly explain each component of the architectural framework.

### 2.1. Sensing Strategies

Ongoing researches of bio-chemical and electro-mechanical sensor technologies have led to a wide range of wearable and implantable sensors, suitable for continuous monitoring. In general, bio-medical sensors can be classified into physiological and bio-chemical sensors based on the medical parameters to be monitored for specific medical conditions [3]. Some of these parameters that are monitored for different medical conditions are listed in Table 3. Important requirements for physiological and biochemical sensor types are sensitivity, selectivity, reliability, ease-of-use, sensor packaging, bio-compatibility, and power consumption. Implantable bio-sensors are often affected by noise due to bio-fouling, motion artifact, and interference. The new sensor designs are focused on reducing these artifacts, thereby increasing the implantability of the sensors.

**Figure 3:**
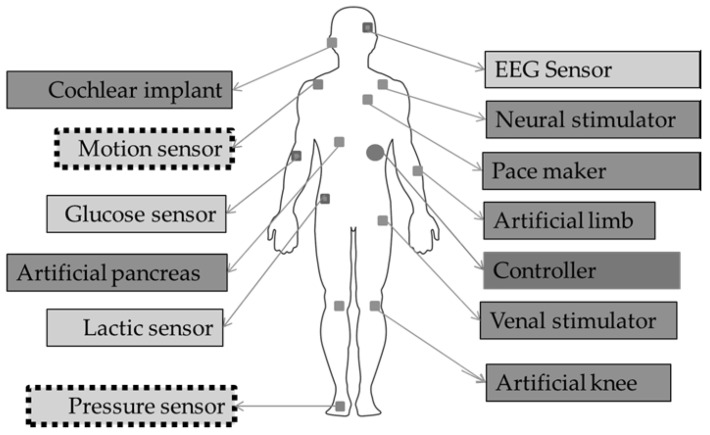
An example of heterogeneity of nodes in IBSN.

The IBSN system should be able to handle the diversity of the sensors without jeopardizing the quality of the sensor data which may result in the misdiagnosis. Figure 3, represents the diversity of the bio-sensors that can be used in IBSN. These medical sensors output different types of medical data at different rates. An ideal network should be self-adaptive to accommodate different kinds of sensor devices. Continuous sensing will increase the power consumption of the sensor node. However, discontinuous sensing can lead to missing life-critical data. A sensing strategy should be able to handle the trade-off between the power consumption and the reliability of the sensing system efficiently.

### 2.2. Actuation Strategies

The actuation in IBSN refers to the medical therapy provided by the medical implants, such as a cardiac pace-maker, neural stimulators, and drug-delivery devices. In most case, actuation is pre-programmed. Similar to a typical sensor node, actuation devices in IBSN usually consist of three fundamental blocks, i.e., an energy source, a micro-controller, and a communication module. In addition to these blocks, bio-medical devices will have an additional block responsible for delivering the therapy.

In the context of a closed-loop medical system, these actuation devices should be programmed based on the feedback from the implanted sensor nodes. In an IBSN, these actuation devices have the highest priority. The medical devices will have dynamic requirements for wireless communication and these requirements are patient-centric. The requirements of different implantable medical actuation devices are listed in the Table 4. The actuation strategy must be adaptive to the requirements of the patient-centric closed-loop medical devices.

### 2.3. Power Scavenging and Energy Efficiency

Power source is one of the key elements of IBSN. It often dominates the size and lifetime of the sensor nodes. Thus far, battery remains the main source of energy for the medical sensor nodes. In parallel to battery source, power scavenging can be used to prolong the lifetime of the sensor and to enable long-term monitoring of the patient. A number of power scavenging sources have currently been proposed, which include motion, vibration, air-flow, temperature difference, ambient electromagnetic fields, light, and infra-red radiation. For instance, Mitcheson et al. developed a vibration-based generator designed for implantable devices, which is capable of delivering 2 μJ/cycle [17]. Similar vibration-based thin film piezoelectric energy scavenging systems were proposed by Reilly et al. [18]. A thermoelectric effect energy-harvester called Micropelt was reported by Böttner et al. [19], which can produce up to 0.6 mW on an area of 5 ${\mathrm{mm}}^{2}$. This is a promising option to be used in medical implants for a long-term operation. The power scavenging is an intermittent source of energy. A battery should be used to store the minimal energy needed for an emergency communication, apart from the routine communication. All other components of IBSN architecture will depend on the power scavenging. Energy-efficiency should be guaranteed by algorithms which will adapt the power usage of all components of IBSN without compromising the prime objective of the medical systems.

### 2.4. Data Handling Strategies

Bio-sensors are often affected by noise due to bio-fouling, motion artifact, and interference. For example, ECG (Electrocardiogram) sensors are highly sensitive to motion artifact, which can hinder its ubiquitous use. To improve the sensor reliability, multi-sensor or sensor array approaches are commonly used [20]. Sensor fusion techniques can then be applied to fuse information from these sensors. In practice, feature selection techniques can be employed to identify relevant vital signatures from an optimum set of physiological data. However, a large amount of raw data is often required for processing. Transmission of this large amount of data will reduce the battery-life significantly when it is done continuously. On-node signal processing or pre-processing of the data within the capability of the sensor node can reduce the active duty-cycle of the wireless communication and enable the transmission of only the critical data to the base station. These on-node processing activities will reduce the amount of data transmitted and result in a significant reduction of power consumption. Distributed processing of data will improve the total network lifetime. They will also enable faster recognition of medical anomalies, improving the accuracy of medical therapies. Special attention should be given to maintaining a low false-positive anomaly detection. However, prolonged and heavy computational processing can cause over-heating problems for the sensor nodes, which can cause fatal damages to the tissues surrounding the implant. For example, in [21], authors theoretically calculated that, 2 °C temperature rise in 1 ${\mathrm{cm}}^{2}$ of tissue, when a neural amplifier was operating at 40 mW power, is valid for most of the tissues in the human body. This variation in temperature was observed for 7 weeks, which resulted in the formation of a fibrous capsule with increased capillary density around the implant. Authors also demonstrated in [21], that the thermal over-load can be minimized by distributing the processing functionality among different devices. It has to be noted that the poor data handling strategies will significantly reduce the reliability of the system. Algorithms which enable not only low-power and less complex operation but also ensure reliability of the system are yet to be designed.

### 2.5. Communication Strategies

Communication strategies deal with the issues as described in the following sections.

#### 2.5.1. Medium of Communication

Communication between the nodes in IBSN can happen in three different manners, namely, in-body to in-body, in-body to on-body, in-body to off-body (a base station). Unlike the Body Area Network (BAN) and Personal Area Network (PAN), communication in IBSN happens between the implanted nodes to a base station placed outside or on the human body. Moreover, BAN and PAN networks have limited use of the latest implantable medical devices, such as deep brain stimulators. This is partly due to the limited capabilities of BAN and PAN, which cannot support the requirements of implantable medical devices in terms of power-consumption, reliability, and quality-of-service parameters [22].

International regulation of the frequency band for life-critical medical devices will benefit the user, the wireless medical industry, and regulators. It will also positively impact on cost-saving, quality, reliability and delivery of health-care. Medical Implant Communication Service (MICS) is accepted globally for the use of medical devices and is standardized [23]. To this end, an IEEE 802.15.6 task group was set up to standardize the in-body communication together with the off-body and on-body communication. One of the main aspects of this standard is to standardize the physical layer with three different radio configurations, namely narrow band, ultra-wide band, and human body communications.

Out of the three radio configurations, MICS band (under the narrow band) was dedicated to the in-body communication, which operates at 402–405 MHz with 10 channels of 300 KHz bandwidth. Body-worn applications are not allowed in the MICS band [22]. The 402–405 MHz frequencies have propagation characteristics conductive to the transmission of radio signals within the human body. Also, tissue absorption is reduced by using a lower transmitting frequency, which has two advantages, one being to improve signal propagation within human body, the other being to reduce the thermal overload caused to the surrounding tissues due to the absorption of radio waves [24].

#### 2.5.2. Medium Access Mechanisms

As mentioned earlier, the wireless radio is the most power consuming block of the sensor node. Ideally, turning on the radio should result in a successful data transfer in order to achieve 100% energy-efficiency and reliability. However, IBSN faces interference in the radio channel, collision, over-hearing of data, and packet-errors. Access mechanisms enable the nodes to efficiently use the medium by eliminating all these problems. The communication strategies of the IBSN should handle all aspects of wireless communication in compliance to the international rules of the wireless spectrum.

#### 2.5.3. Network Topology

The topology of IBSN can be in the following forms:

- Star topology: A star topology-based IBSN consists of a central controller (namely the coordinator), which initiates, terminates, and manages the transmission within the network. The communication in a star topology network is either between the coordinator and device (downlink) or between device and the coordinator (uplink). Note that the peer-to-peer communication (device-to-device) is not considered here. The coordinator uses beacon commands to identify and manage (such as create, maintain and terminate) communication in an IBSN.
- Cluster-tree topology: The cluster-tree topology is a type of a multi-hop mesh network, in which there is always only one single path between two devices. The first device starting the network becomes the root of the tree. Another device can join the network as a “child” of the root node. It in turn allows other devices to join the network. Devices are aware of their “parent” node and any “child” nodes. This hierarchical topology reduces routing complexity. An advantage of the cluster-tree approach is that it enables low power consumption of leaf nodes which, in the case of IBSN, can be the implanted life-critical nodes.
- Star-mesh hybrid topology: This topology allows the connection of a mesh network with one or more star networks or several star networks with each other. A mixed star and mesh network topology combines the simplicity of the single-hop star topology with the scalability and flexibility of the multi-hop mesh topology.

#### 2.5.4. Routing

Routing within IBSN is plain since the number of nodes in the network is less and topology is simpler than conventional WSN. However, the physical channel is highly dynamic due to human behavior and rapid changing environment. A good routing mechanism must be able to handle the dynamic changes of the link quality between the nodes in compliance with the IEEE 802.15.6 standard of a physical layer.

#### 2.5.5. Security

Security is paramount in IBSN as the wireless transfer mainly includes life-critical data and any interference to the data can cause fatalities. IEEE 802.15.6 standard presents a strict guideline for authentication of devices complying with level 2 security in the data-link layer [23]. Innovative security keys which use bio-signals for encryption and authentication are available, however the limited resources of IBSN make the implementation of security mechanisms challenging.

## 3. Design Challenges of MAC Protocol for IBSN

The design of an efficient MAC protocol for IBSN faces a number of challenges, such as interoperability, scalability, QoS, and energy efficient communication. There are various low-power techniques, such as the fixed duty cycling technique in SMAC [25] and adaptive wake-up slots in TDMA [26] to ensure energy efficient communication in a wireless sensor network. They are, however, not energy-efficient for heterogeneous IBSN and will not suffice. Unlike the supported scenarios by SMAC, the traffic characteristics in a IBSN vary from periodic to non-periodic and vice-versa [8]. Therefore, the concept of the fixed duty cycling technique gives limited answers when it comes to the heterogeneous behavior of autonomous sensor nodes in a IBSN. The dynamic nature of these nodes does not urge synchronized periodic wake-up periods. Data from some of the sensor nodes, for instance, electrocardiogram (ECG), may be set at a one packet per hour rate to the coordinator, while others may send data twice a week. Nodes should also have the capabilities to sense and transmit emergency information. The data traffic in IBSN is either normal, on-demand, or burst.

### 3.1. Requirements of MAC Protocol Design for IBSN

The fundamental task of a MAC protocol is to avoid collision of data packets and to prevent simultaneous transmissions while preserving maximum throughput, minimum latency, communication reliability and maximum energy-efficiency [25]. QoS is also an important factor representing a good MAC protocol. In medical applications, a latency of only 125 ms is allowed, whereas in consumer electronics latency can be less than 250 ms [7]. Other important features include adaptability to a dynamic physical medium, maximum achievable throughput in different network scenarios, low jitter in heterogeneous traffic, efficient bandwidth utilization with high payload, safety and security. Table 5 presents major features of MAC protocols for IBSN and their values suggested by the IEEE 802.15.6 [23]. In general, a good BSN MAC protocol should be energy-efficient, reliable even in the presence of heterogeneous traffic, safe and secure, in addition to being QoS-aware [27]. The closed-loop mode of operation of medical devices does not only require these features but also additional features, such as ability to intelligently access the medium in case of emergency events, establishing reliable and low latency communication between nodes and medical devices, and ability to respond quickly within the specific time-frame for a given medical device.

The features of the MAC protocol specified by the IEEE 802.15.6 are common for the in-body and on-body BSNs, however they need to be better specified to suit the requirements of closed-loop medical devices.

#### 3.1.1. Energy-Efficiency

To deliver the levels of comfort and unobtrusiveness required for widespread adoption, IBSN sensor nodes must be small and have energy sources that last for months and years, depending on the application. The size requirement obviously limits the size of the batteries that will power the nodes (energy scavenging is another option, but the amount of power available from such techniques is relatively small), so IBSN nodes must be extremely efficient in their energy usage. The IEEE 802.15.6 standard recommends a star topology, in which the coordinator node is rechargeable and is placed outside the body. Most network management activities, such as time slot allocation, routing information, and prioritization are performed by the coordinator node. By doing so, energy wastage, due to performing such management overheads by the client nodes, is minimized. However, in some of the closed-loop applications of IBSN, the implanted client nodes are also responsible for such management activities in order to provide high reliability [8]. Furthermore, energy efficiency is also increased by mechanisms that allow sensor nodes to enter a low-power sleep mode for a long time (several beacon periods) before transmitting/receiving. Low power WuR is required to achieve such long sleep duration without affecting the reliability.

#### 3.1.2. Reliability

Reliability in wireless communication refers to the number of successful receptions of packets and the number of packets transmitted through a wireless link between two nodes. The wireless link between two nodes in IBSN is highly deteriorated by the conductive human tissues and dynamic human activities. The MAC protocol should have additional mechanisms, such as acknowledgment schemes, and beacon synchronization schemes to overcome the dynamic link quality and to increase the reliability [28]. Enhanced reliability, such as integrity and authenticity of the data packets, is achieved by adding redundant information bits in the form of a cyclic redundancy check (CRC) and message integrity code (MIC) in the IEEE 802.15.6 standard. Thus, there is generally a trade-off between a higher reliability and throughput. Priority nodes sending medical data should be ensured of reliability as it contains life-critical information in IBSN. An efficient MAC protocol will have an optimum reliability by adapting to the application scenarios and the context of data.

#### 3.1.3. Overhead

Prior to the communication of useful data, the sensor nodes need to exchange the management packets between each other. The efficient management of packets will ensure that all sensor nodes are aware of other sensor nodes’ resources, schedules of transmission and reception, channel availability information and general information about useful data packets that are to be transmitted. Management of packets not only creates overheads but also increases the active duty-cycle of the sensor nodes. IEEE 802.15.6 requires a large duty-cycle range: from 0.1% to 30%. Medium access mechanisms need to be adaptive in terms of their management, access, and communication strategies to meet the duty-cycle requirements of a variety of medical devices. It is important to note that the duty-cycle is set by the spectrum regulating institutions, which limits the the duty-cycle of the MICS band to 0.01% i.e., approximately 36 s of access to the medium in an hour [23]. Alternatively, the regulations allow the nodes to communicate with a *listen-before-talk* mechanism, preventing the nodes from communicating if the physical medium is already occupied by some other device. In this aspect, the amount of overhead data required for link and network management should be minimal and not higher than the actual useful data to be transmitted or received.

#### 3.1.4. Throughput

Network throughput is measured by average number of received data bits per second. Packets can be lost due to loss of connectivity or collisions, among others. In the MICS band, the radio channel has been shown to have an essentially slow data-rate. Although flat-fading and inter-symbol interference from multi-path are insignificant in the MICS frequency band, low power transmission, and dynamic link quality due to human activities deteriorate the effective throughput of the network. Heterogeneous medical devices in IBSN demand dynamic throughput for different applications. For example, an EEG sensor requires a much larger throughput (i.e., in the orders of 150 Kbps), whereas glucose sensors require a much lower throughput (e.g., in the orders of 30 Kbps). MAC protocols should be able to operate efficiently in spite of the restrictions set by the spectrum regulations and at the same time meet the dynamic requirements of heterogeneous sensor nodes. Existing MAC protocols are not specifically designed to address such complex application scenarios. Our analysis in Section 4 describes why existing MAC protocols do not meet the requirements of IBSN.

#### 3.1.5. Latency

Latency is the delay between sending and receiving data across any two nodes. A heterogeneous IBSN has different medical applications that have a wide range of latency requirements. For example, in the case of DBS applications, 100 ms of latency is strictly required, whereas in long-term monitoring applications, such as cardiac monitoring, latency up to 1 s is tolerated. The latency is to be increased when the data is hopped through a relay node between transmitter and receiver. However, IEEE 802.15.6 suggests that the relay node should account for achieving the overall latency of the communication. A MAC protocol is required to accommodate the relay node and also should be able to achieve the latency required by the medical application. For life-critical medical nodes, achieving both energy efficiency maximization and latency minimization is difficult. A complementary solution needs to be developed instead of resorting only to the regular MAC frame structure.

#### 3.1.6. Hardware Complexity

IBSN sensor nodes have much less resources than conventional sensor networks due to their strict size restrictions and bio-compatibility. In such cases, introducing additional hardware only to increase the performance of wireless communication is not an option. However, with the advent of nano-technology and system-on-chip design, the addition of minimal hardware components, such as a wake-radio has been made possible without increasing the total surface area of the chip itself [14]. Although the addition of minimal hardware is a viable option, it will have consequences in terms of energy consumption and may even affect performance of the wireless communication.

### 3.2. Types of Access Mechanisms Recommended for IBSN

The MAC protocols applicable to IBSN are broadly divided into the following categories:

- Time Division Multiple Access Mechanisms (TDMA)
- Carrier Sense Multiple Access Mechanisms (CSMA)
- Hybrid Access Mechanisms
- Random Access Mechanisms

Each access mechanism has its own advantages and disadvantages. The basic principle of each mechanism is further explained below.

#### 3.2.1. Time Division Multiple Access (TDMA)

The scheduled access mechanism of the physical medium is carried out either by scheduling the content or carrier. In TDMA-based approaches, scheduled access is carried out by synchronizing all nodes in the network to a specific time-frame and allocating a scheduled time slot for each sensor node. The advantage of this method is enabling collision-free communication due to time schedules being set up. This will reduce the power consumption and increase the throughput of the network. However, the time schedule needs an accurate time synchronization between the nodes. A deviation in the synchronization will cause the network to operate less efficiently or sometimes even fail to operate. TDMA is considered to be a suitable candidate for the IBSN for the following reasons:

- Time synchronization is less complex due to the smaller size of the IBSN compared to other WSN applications.
- The star topology is preferred for IBSN where a central network controller (CNC) is always present outside the body in close proximity of the network. This enables simpler coordination between the nodes.
- Collision avoidance is easier with low power consumption.

#### 3.2.2. Carrier Sense Multiple Access (CSMA)

The random access of the physical medium is carried out by Carrier Sense Multiple Access/Collision Avoidance (CSMA/CA). In contrast to the IEEE 802.3 standard, which uses collision detection, the IEEE 802.15 (wireless medium) standard uses collision avoidance in order to save some power. The collision avoidance is performed by setting a back-off counter to a random integer in the range of the maximum allowed contention window (CW), uniformly distributed over the interval [1,CW] where $CW\in (CW_{min},CW_{max})$. Performance of CSMA/CA in terms of throughput and latency depends largely on the number of sensor nodes present in a network and the amount of data being communicated. If a node has a large amount of data, it uses the physical channel to send the data, consequently preventing other nodes from gaining access to the medium. This can result in very low reliability for the nodes trying to send high priority medical data. Also, most of the energy is wasted in idle listening, as the nodes have to listen to the medium for the data packets destined for them. A pure CSMA/CA technique has several limitations, including high energy-consumption, high latency, and very low reliability. However, various hybrid CSMA/CA protocols have been developed for BSN applications including health monitoring. Performance of these hybrid CSMA/CA protocols can be improved by incorporating techniques such as scheduling the random access in dedicated time-slots, prioritizing the sensor nodes, and incorporating WuR to wake-up specific sensor nodes before sending data.

#### 3.2.3. Hybrid Access

The hybrid access mechanism uses CSMA for non-realtime data communication and TDMA for real-time data communication. The data is sent in time-slots based on the priority of the nodes generating data. The time slots can be set depending on the application. The main disadvantages of the hybrid access protocols are resource exhaustion of the client nodes, high energy consumption, and longer beacon-periods for the dynamic allocation of time-slots.

Frequency Division Multiple Access Frequency Division Multiple Access (FDMA) is a complex hybrid access mechanism, in which a full frame of frequency band is made available for communication. This results in the continuous flow of data between the sensor nodes, which improves efficiency of transmission. In FDMA, each channel is assigned to only one node at a time. Each node shares a channel simultaneously, though transmits at single frequency. FDMA is used with both analog and digital signals [29]. It requires high-performing filters in radio hardware, in contrast to TDMA and CSMA. As each node is separated by its frequency, minimization of interference between nodes is done by sharp filters.

ALOHA is a random access technique in which, whenever a packet is generated, it is transmitted immediately without any further delay. Successful reception of a packet depends only on whether it is collided or not with other packets. At the end of packet transmission, each user knows whether its transmission has been successful or not. If collision occurs, the user schedules its re-transmission to a random time. The randomness is to ensure that the same packets do not collide repeatedly. In the ALOHA technique, the node checks for the availability of data packets to be transmitted. If they are available, then the node transmits them, otherwise the process ends.

Slotted ALOHA is a variant of Pure ALOHA with a channel that is divided into slots. Restriction is imposed on users to start transmission on slot boundaries only. Whenever packets collide, they overlap completely instead of partially. So only a fraction of slots in which the packet is collided is scheduled for re-transmission. It almost doubles the efficiency of slotted ALOHA as compared to pure ALOHA. Successful transmission depends on the condition that only one packet is transmitted in each frame. If no packet is transmitted in a slot, then the slot is idle. Slotted Aloha requires synchronization between nodes which lead to its disadvantage.

The hybrid access is not optimized in terms of energy efficiency but it is optimized in terms of high throughput and reliable packet delivery.

#### 3.2.4. Adaptive Access Mechanisms

Adaptive access mechanisms are improvised hybrid access mechanisms, which are adaptive to the dynamic changes of the system. The underlying working principle of them is based on either contention-free access, contention-based access or both. Unlike hybrid access mechanisms, adaptive access mechanisms incorporate learning techniques to adapt the underlying access mechanisms, based on continuous feedback from the system. For example, consider a simple frame-based contention access mechanism, in which each node determines its own wake-up schedule based on the real-time requirements of the node. Such a non-fixed adaptive duty-cycle not only improves energy-efficiency, but also improves network performances over time. The node learns about the flow of data over time, and using learning algorithms, the node can teach itself to derive an optimum schedule of duty-cycles. Also, in the case of IBSN, the dynamicity in the flow of data can be handled easily with adaptive access mechanisms. However, these adaptive access mechanisms are still in their infancy, where learning algorithms require large memory and computing power to operate. In IBSN, the amount of resources is stringent, which widely limited its direct application in MAC protocols for IBSN.

## 4. Taxonomy of MAC Protocols Based on Their Use of WuR Technology

**Figure 4:**
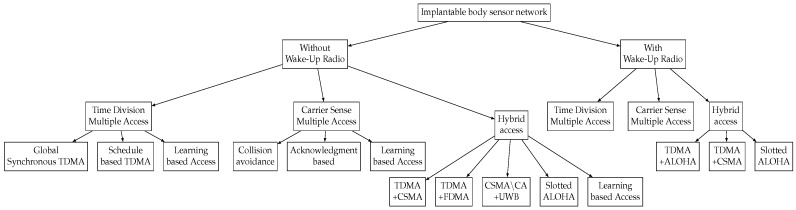
Taxonomy of MAC protocols based on their use of WuR technology.

The WuR is a new technology which is specifically used to improve energy-efficiency of MAC protocols. The IEEE 802.15.6 standard suggests the use of WuR-based communication in medical implants. In this context, we classify the existing MAC protocols based on their use of WuR technology. The taxonomy of the access mechanisms based on their use of WuR technology is presented in Figure 4. One should note that, to the best of our knowledge, no MAC protocol has been so far designed specifically for IBSN. However, some of the crucial techniques used in these protocols, such as adaptive sleep mode, and cross-layered access control may meet the requirements of IBSN. Therefore, after providing a brief introduction to them, we study their applicability for IBSN and their ability to meet IBSN requirements. Table 6, Table 7, Table 8, Table 9 and Table 10 present our analysis of the advantages and disadvantages of these protocols for IBSN applications.

### 4.1. MAC Protocols without WuR

MAC protocols that do not make use of WuR can be classified into the following groups of access mechanisms.

#### 4.1.1. Time Division Multiple Access (TDMA)-Based MAC Protocols for IBSN

Marinkovic et al. [30] present an energy-efficient low duty-cycle MAC protocol that enables access to the physical layer for a hierarchical topology consisting of nodes communicating with master nodes, which in turn communicate with the monitoring station. The hierarchy removes the need for sensors to expend power by transmitting to the monitoring station. Also, the use of TDMA ensures collision-free transfer and minimization of idle-listening. The protocol is implemented using the ADF70XXMBZ2 platform with ADF7020 RF transceivers, manufactured by Analog Devices, Inc., USA. Measurements reveal that the protocol is energy-efficient for streaming and short-burst data communications. A novel TDMA-based protocol for BSNs, called H-MAC, is presented in [34]. This protocol improves energy-efficiency by using the heartbeat rhythm to perform TDMA synchronization, avoiding energy consumption associated with transmitting time synchronization beacons. Power efficiency is also guaranteed in H-MAC as a TDMA-based protocol assigns time slots to each bio-sensor to guarantee collision-free transmission. Simulations show that H-MAC prolongs the network life of sensors dramatically. In [36], a TDMA-based MAC protocol called BodyMAC is proposed. Three types of bandwidth allocation schemes are devised to cope with different types of data communications, such as periodic data sensing and important event allocation. In conjunction with bandwidth allocation, a sleep mode mechanism is introduced, which turns off a node’s radio during beacon, up-link and down-link periods, as much as possible. Simulation results show superior performance of BodyMAC compared to that of IEEE 802.15.4 MAC. Timmons et al. [27] introduce an adaptive TDMA-based MAC protocol called MedMAC. MedMAC incorporates a novel adaptive TDMA synchronization mechanism in which only a multi-superframe beacon has to be listened to by the nodes. An optional contention period is also available for low-grade data, emergency operation and network initialization procedures. Simulations show that MedMAC consumes less power than IEEE 802.15.4 for two classes of medical applications. In [69], a power efficient MAC protocol is proposed for WBANs. This work presented a traffic-based wake-up mechanism that utilizes the three categories of traffic patterns of the body sensor nodes, namely normal traffic, on-demand traffic and emergency traffic. The wake-up patterns of all body sensor nodes are organized into a table called traffic-based wake-up table. The table is maintained and modified by a network coordinator according to the application requirements. Based on the body sensor node’s wake-up patterns, the network coordinator can also calculate its own wake-up pattern. During normal traffic, both the body sensor nodes and the network coordinator send data based on the traffic–based wake-up table. A MAC protocol for static BAN is introduced in [32]. A Master Node (MN) collects data from body nodes and communicates with a Monitoring Station (MS). Received data is analyzed by a MS while the on-body network coordination and synchronization is performed by a MN. Time slots $S_{1}$ to $S_{n}$ are allocated to sensor nodes while time slots $R_{S1}$ to $R_{S2}$ are reserved which are assigned when requested. The number of these extra time slots depends upon targeted packet drop, packet error rate and number of sensor nodes.

#### 4.1.2. Carrier Sense Multiple Access (CSMA)-Based MAC Protocols for IBSN

Authors in [40] presented B-MAC protocol which renders typical properties of a IBSN, such as simple implementation on hardware, highly predictable performance parameters, and tolerance to network changes. B-MAC implements an asynchronous access mechanism similar to X-MAC. B-MAC has a relatively high reliable data packet delivery ratio of 98.5% [41]. B-MAC could be optimized for star topology. However, B-MAC also suffers from hardware constraints such as memory and computational overhead. In [44], Huq et al. present MEB-MAC, which focuses on the channel access delay reduction for medical emergency traffic with high reliability. No energy efficiency is concerned with the implementation and analysis. It has an adverse effect on new node insertion and mobility of the network. An energy efficient MAC protocol called O-MAC is presented in [45]. O-MAC has achieved increased energy efficiency by novel receiver scheduling methods, such as Staggered On and Pseudo-randomized Staggered On. Theoretical analysis and practical implementation reveals that the protocol is 70% more energy efficient than B-MAC, S-MAC and T-MAC. The CSMA-based MAC protocol presented in [43] is called DISSense. A good analysis of MAC problems in different typologies and the benefits of cross-layer optimization are addressed in this paper. Features such as data delivery ratio, latency, duty cycling and adaptability are better than other similar protocols. DISSense can achieve good QoS in small scale networks and proportionately increase with network size. However, the performance of DISSense is traded off with energy consumption. No clear analysis of energy-efficiency is carried out though.

#### 4.1.3. Hybrid Access Based MAC Protocols for IBSN

Otal et al. [47] proposed an energy-saving MAC protocol, called DQBAN for BAN as an add-on to the 802.15.6 MAC protocol which suffers from low reliability and limited QoS in real-time environments. The proposed DQBAN is a combination of a cross-layer fuzzy-logic scheduler and energy-aware radio-activation policies. The queuing of access packets and data packets is determined by fuzzy-logic rules, which permit body sensors to find out ‘how favorable’ or ‘how critical’ their situation is in a given time-frame. The logic scheduling MAC protocol optimizing QoS and energy consumption by considering cross-layer parameters, such as residual battery lifetime, physical layer quality and system wait time, is presented. The authors tested their proposed protocol on two scenarios: a homogeneous scenario of a body sensor network with 5–35 homogeneous ECG wireless sensors and a heterogeneous scenario of a body sensor network with wireless ECG sensors and four other sensors for clinical doctor PDA, respiratory rate, blood pressure and endoscope imaging.

In [59], the authors proposed the use of a UWB transmitter for energy-efficient operation of WBANs. Due to the high interference generated by the human body and its environment, one solution to develop low power-output transceivers for radios in the sensor nodes is to optimize the air interface of the network. By creating architectures that exploit features of robust nodes and energy-constrained nodes, low latency and simple network topologies, the authors show that an UWB-based architecture is advantageous over narrowband radio communication.

In [57], the slotted ALOHA is employed in the contention access period (CAP) to request the slot allocation. A mini-slot method is designed to enhance the efficiency of the contention. Moreover, sufficient slot allocation in the contention-free period (CFP) enables adaptability to different traffic load conditions.

#### 4.1.4. Adaptive Access Based MAC Protocols for IBSN

Galzarano et al. [52] proposed an adaptive access mechanism called QL-MAC based on the Q-learning technique. In QL-MAC, a simple asynchronous CSMA-CA approach is employed over a frame-based structure. It basically divides the time into discrete time units, i.e., the frames, which are further divided into smaller time units, i.e., the slots. Both frame length and slot number are parameters of the algorithm and remain unchanged at the execution time. By means of a Q-Learning-based algorithm, each node independently determines an efficient wake-up schedule in order to limit, as much as possible, the number of slots in which the radio is turned on. Such a non-fixed and adaptive duty-cycle reduces the energy consumption over the time without affecting the other network performances. Authors of [70] compared the performance of QL-MAC with the performance of conventional MAC protocols, such as S-MAC [25] and T-MAC [67] in an OMNETT-based simulation environment. The results show that the adaptive behavior of QL-MAC guarantees better network performances with respect to both the packet delivery ratio and the average energy consumption.

A similar approach is followed in a reinforced-learning-based medium access control protocol called RL-MAC [53]. The distinct feature of the protocol is that both the frame active time and duty-cycle are dynamically modified in accordance with the node’s traffic load as well as its incoming traffic characteristics. The learning algorithm is focused on maximizing energy-efficiency and throughput.

### 4.2. MAC Protocols with WuR

A TDMA-based scheme combined with WuR can be used to design a power efficient MAC. Authors of [28,60,61], have proposed TDMA-based MAC protocols for IBSN with WuR. Use of WuR can minimize the extra power consumption by the RTS–CTS packet exchange which is done by the main radio. The packet inter-arrival parameters are re-configurable values for each node. For example, in the case of a patient, a doctor/nurse or in-charge person can set the packet inter-arrival time for temperature monitor to be 6 h or 21,600 s. This will cause the central node controller (CNC) to send a WuR signal to the particular node after the specified intervals and complete the data communication. The node, between two consecutive wake-up periods, can switch off its main radio and go to the sleep state to save power. It does not have to contend for data communication.

An ultra-low-power and traffic adaptive protocol designed for BAN is discussed in [71]. They used a traffic adaptive mechanism to accommodate on-demand and emergency traffic through wake-up radio. Authors of [60] have proposed a MAC protocol which supports dependability and QoS guarantee for the most important life-critical message and majority of real-time traffic. The protocol can be used with different physical layers: UWB, MICS, WMTS, HBC. A dynamic network size greater than six nodes to less than 100 nodes per network can be achieved. An improved quality-of-service addresses throughput, access latency, and priority . High scalability is realized. Star, cluster-tree and peer-to-peer, are supported in the MAC protocol proposed in [61]. Table 10 comprehends the MAC protocols with WuR based on the special feature and potential drawback with respect to the IBSN scenario.

## 5. Comparison of MAC Protocols for IBSN

We considered more than 30 MAC protocols designed for generic sensor networks and BSN in order to broadly evaluate their use in IBSN. Table 11 is a qualitative comparison of different protocols based on network features discussed in Section 3. We used the results of each protocol as mentioned in the respective article to qualitatively analyze the trade-off made between each requirement, such as energy-efficiency, reliability, software overheads, throughput, latency, and hardware complexity. This comparison will present the commonly made trade-offs in the MAC protocols designed for BSN and WSN.

Energy efficiency is the amount of energy consumed to successfully transmit a data packet including the energy consumed by the network management overheads. (+) for energy efficiency indicates that the MAC protocol is specifically designed to operate at low power application, such as BSN. It also indicates that the main aim of the protocol was to improve energy-efficiency and longer network life-time over the performance metrics such as throughput, reliability and QoS. (−) indicates that the MAC protocol consumes an enormous amount of energy for successful communication, which can be caused due to large overheads, elongated active duty-cycle, or lack of collision-prevention mechanism. (*O*) indicates that the MAC protocol was not designed to be energy-efficient, but rather to improve the performance metrics such as throughput, reliability and other QoS.

Reliability is the successful data transfer from the transmitting node to the receiving node. (+) for reliability indicates that the MAC protocol was designed to deliver reliability even in a dynamic physical medium, such as the human body environment, where the link quality is highly deteriorated both temporally and spatially. (−) indicates that the MAC protocol compromised reliability to improve other parameters, such as energy-efficiency and throughput. (*O*) indicates that the MAC protocol was not designed to provide reliability in highly dynamic link quality.

Overhead is the amount of additional data that is required apart from the payload to successfully establish a link between the transmitting node and the receiving node. (+) for software overhead indicates that the MAC protocol aims at minimal management phase data communication between the coordinator and still achieves better performance in terms of throughput and reliability. (−) indicates otherwise. (*O*) indicates that the MAC protocol has no additions in the overhead in relation to the conventional overheads.

Throughput is the amount of information bits received per second at the receiver. (+) for throughput indicates that the main aim of the protocol was to improve the throughput of the network and (−) indicates otherwise. (*O*) indicates that the throughput aspect of the MAC protocol was not explicitly available.

Low latency is the amount of time required to establish a link before any useful data transfer. (+) for latency indicates that the MAC protocol is designed to achieve low latency for each transmission and (−) indicates otherwise. (*O*) indicates that the latency aspect of the MAC protocol was not explicitly available.

Hardware complexity is additional hardware components such as WuR that are required to achieve the intended performance of the MAC protocol. (+) indicates that the MAC protocol requires additional hardware other than the main radio to achieve better performance and (−) indicates otherwise. (*O*) indicates that the latency aspect of the MAC protocol was not explicitly available.

All MAC protocols mentioned in Table 11 are compared based on the requirements of IBSN applications, while none of them are explicitly developed for IBSN applications. Although some of the protocols are developed for general WSN application, they are still considered in our comparison since their features meet the requirements of the IBSN applications.

## 6. Discussion

### 6.1. The Need for WuR

**Figure 5:**
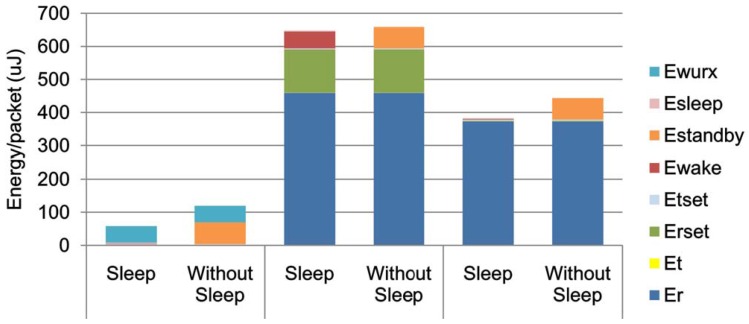
Importance of WuR in IBSN [75].

The operation of WuR is not only simple but also energy efficient. The dual radio architecture is superior because the power consumption of the main communication radio scales up with increasing network traffic. The energy consumption could be reduced significantly when using appropriate wake-up techniques [15]. Consequently, it results in more relaxed synchronization requirements for schedule-based MAC protocols [14]. Based on our previous research, work reported in [8], the power consumption of WuR-based MAC protocol, synchronous MAC protocol and asynchronous random access protocols is compared. It is shown in Figure 5 that the power consumption of WuR is very low compared with the power consumption of scheduled access and random access mechanisms. The ultra-low-power and performance trade-off can be achieved by creating a proper Tx/Rx link-budget for the main radio. The WuR sets a lower-bound of power consumption in idle state compared with the current available technologies which could be mitigated by applying duty-cyling to the WuR.

### 6.2. Research Issues and Challenges

The amount of energy available for the IBSN sensor nodes is scarce and nodes are expected to last in the order of months to years. The MAC protocols, which are commonly designed for wireless sensor networks do not support high and severe quality-of-service requirements of IBSN applications in terms of high throughput, high reliability and low latency. MAC protocols of generic BAN applications focus mainly on monitoring applications. In IBSN, the actuation scenario introduces additional reliability requirements in terms of very low latency and guaranteed packet delivery. In general, existing energy-efficient sensor network applications operate with fixed low duty-cycle. In IBSN scenarios, however, the active duty-cycle is dynamic and may vary through time depending on the medical applications (for example for long-term monitoring of symptoms and time sensitive medical therapy information, such as a cardiac pacemaker).

Although MAC protocols for BAN take into account the body effect on the physical channel, long-term operation and battery life, their use in life-critical medical application is questionable. Significant improvements in MAC protocol to achieve the ultra-low-power consumption are to be made.

To ensure reliability in highly dynamic sensor network topology, MAC protocols may be designed with redundant acknowledgment schemes and extensive link quality assessment, which often are not energy-efficient. Conventional methods to ensure reliability can be improved significantly by complementing it with bio-inspired design, benefiting from the predictable patterns of link deterioration caused by everyday human activities such as walking and running. Furthermore, reliability can be improved in an energy-efficient manner by utilizing bio-signals for synchronization and acknowledgment purposes.

Generally, MAC protocols of BSN are designed to support devices which are intended only for sensing operation. In IBSN, nodes can also be actuation devices which have different characteristics to sensing devices. In such cases, the MAC protocol should be able to provide the QoS support for actuation and sensing devices to support heterogeneous data communication. Apart from priority, these medical actuation devices also require real-time communication to accurately prognose and diagnose the medical condition with strict time constraints.

Although existing MAC protocols for BSN are largely adaptable to different environmental and sensing conditions. They are however not adaptable to the dynamic changes required in the flow of information within the IBSN for different medical applications. For example, in the complex closed-loop operation of DBS for epileptic patients, predicting the onset of seizure requires input from different bio-sensors within the network at different time stamps. A MAC protocol designed for long-term monitoring applications of BAN cannot adapt to such complex requirements of the closed-loop medical devices. The level of adaptability in existing MAC protocols is very limited. For example, in hybrid access mechanisms, either time-slots are adaptive or the contention window is adaptive according to the traffic load. In dynamic networks, such as IBSN, more than one dimension of the MAC aspect has to be adaptive.

Conventional MAC protocols of BAN are streamlined to send data to the physical medium as soon as possible. In some protocols, the link quality is predicted at the MAC layer before initiating any communication. This greatly improves the performance of the MAC layer in terms of energy-efficiency. In IBSN, the bio-signals and their characteristics, such as periodicity, can help reduce the synchronization overheads and consequently improve the performance of MAC protocol. Additionally, by knowing the type of bio-signal and the destination node to which its data will be sent, data packets may be prioritized and adapted to increase the reliability.

Adaptive MAC protocols are capable of learning additional information about the type of data that are being communicated. However, the learning algorithms are not designed for medical applications. Accuracy of these learning-based MAC protocols should be demonstrated for medical purposes, and error free implementation of these algorithms is yet to be studied. Incorporating WuR to these adaptive MAC protocols can greatly reduce the power consumption, where nodes can benefit from the low power operation of WuR when establishing newer wake-up schedules. Use of WuR for the learning phase of these adaptive MAC protocols will have significant improvement in terms of energy-efficiency and QoS performance.

Furthermore, co-optimization of MAC and the PHY layer between the main radio and WuR can improve energy-efficiency in dual radio IBSN devices. Incorporating WuR at the MAC level rather than using only the wake-up interrupt will improve the reliability of the data communication.

## 7. Conclusions

In this paper, we analyzed various existing MAC protocols considered suitable for IBSN application. The architectural framework and requirements for developing an IBSN protocol are presented in this work. IEEE 802.15.6 standard recommendations were used to define the requirements for IBSN. Crucial improvements that could be amended to the standard were discussed. The design requirements of the IBSN communication scheme are based on a specific IBSN scenario. The several existing MAC protocols are explored and compared for the given IBSN application scenarios. TDMA and contention-based mechanisms could be used for developing MAC protocols, however, they suffer from synchronization, low reliability, high latency and memory overhead. A comparative study is also done, to identify the most applicable access mechanism for the implant communication. In addition, we presented the research challenges that exist in the design of MAC protocols for implant communication. Based on this study, we recommend the implementation of a collaborative WuR and main communication radio in deploying a MAC protocol for IBSN.

## Figures and Tables

**Table 1:** Differences between the requirements of BSN and IBSN. Inspired from [7].

Characteristics	Body-Worn Sensor Networks	Implantable Body Sensor Networks
Communication range	Up to 50 m	Up to 3 m
Number of nodes	Up to 10 nodes	More than 10 nodes
Node functionality	Non-critical, Entertainment, Relays	Life-Critical
Sensor accuracy	Not very accurate	Very accurate and reliable
Size of the node	Wearable size, but not limited in dimensions and bio-compatibility	Should be implantable and very small compared to BSN
Environment	Outside the body, Electromagnetic properties being influenced by the environment	Inside the body, either shallow or deep implant. Electromagnetic properties vary significantly
Event detection	Events are not life-critical, and detection algorithm can be offline and complex	Events are life-critical and detection algorithm should be simple and online
Heterogeneity	Medium in terms of devices, sensing and actuation capabilities	Medium in terms of devices, sensing and actuation capabilities
Security	Security is required but not critical	Security is crucial since the life-critical operations can be fatal is security is threatened
Energy efficiency	Can be recharged. Hence energy constraints can be relaxed	Replacement of the battery is not an option and lifetime of the nodes is in the order of months to years. It has to be highly energy-efficient
Energy availability	Energy is abundant	Energy is scarce and needs to be used with efficiency
Energy harvesting	Energy harvesting is easier from mechanical energy, solar energy, and recharging is also an option	Energy harvesting is very limited, temperature change in the body, chemical changes from glucose and heart vibrations are possible. However, the amount of energy that can be harvested is much lower than the energy harvested from outside the body
Access to nodes	Can be very easily accessed by people and without the help of doctors	Cannot be accessed without the physician and small surgery
Bio-compatibility	Bio-compatibility is not required	Bio-compatibility is of prime importance, since the nodes are planted inside the body
Context-awareness	Not always required	Required
Wireless medium	Mostly air, and surface of the human body	Complex layers of muscle tissues, bones and conducting heterogeneous medium
Connectivity	Should be connected to the Internet	Primarily connected to the base station placed in close proximity
Duty cycling	Very low	Dynamic depending on the application
Interference	Shared with ISM band	Dedicated frequency band for medical applications

**Table 2:** Features of closed-loop medical systems. Inspired from [7].

Characteristics of Closed-Loop Medical Systems	
Self management	A closed-loop system needs to have detailed knowledge about its components, current status, ultimate capacity, and all connections to other systems to govern itself through effective resource management, utilization and sharing
Self configuration	A closed-loop system should automatically and dynamically configure and reconfigure itself under varying conditions and changing environments
Self optimization	A closed-loop system should constantly optimize its performance and resource utilization by monitoring its constituent components and fine tune work-flow to achieve predetermined performance and resource utilization goals
Self healing	A closed-loop system should gracefully recover from routine and extraordinary events that cause component malfunction. It is able to discover problems and establish means of using alternative resources or configurations to maintain system functionality
Self protection	A closed-loop system must be able to exert self-protection by automatically detecting and identifying different types of attacks to maintain overall system security and integrity
Self adaptation	A closed-loop system must be context aware and adapt itself for improved interaction and performance under changing working environments and user requirements
Self integration	A closed-loop system should fully function under heterogeneous infrastructure and be seamlessly and securely integrated with other systems
Self scaling	A closed-loop system should anticipate the optimized resources required and scale its functionality while keeping its complexity hidden from the user

**Table 3:** Parameters to be monitored for different medical conditions. Inspired from [7].

Disease Process	Physiological Parameter (Sensor Type)	Biochemical Parameter (Sensor Type)
Hypertension	Blood Pressure (implantable/wearable) mechanoreceptor	Adrenocorticosteroids (implantable biosensor)
Ischaemic Heart Disease	Electrocardiogram (ECG), cardiac output (implantable/wearable ECG sensor)	Troponin, creatine kinase (implantable biosensor)
Cardiac Arrhythmia/ Heart Failure	Heart rate, blood pressure, ECG, cardiac output (implantable/wearable mechanoreceptor and ECG sensor)	Troponin, creatine kinase (implantable biosensor)
Cancer (Breast, Prostate, Lung, Colon)	Weight loss (body fat sensor) (implantable/wearable mechanoreceptor)	Tumor markers, blood detection, nutritional albumin (implantable biosensor)
Asthma/COPD	Respiration, peak expiratory flow, oxygen saturation (implantable/wearable mechanoreceptor)	Oxygen partial pressure (implantable/wearable optical sensor, implantable biosensor)
Parkinson’s Disease	Gait, tremor, muscle tone, activity (wearable EEG, accelerometer, gyroscope)	Brain dopamine level (implantable biosensor)
Alzheimer’s Disease	Activity, memory, orientation, cognition (wearable accelerometer, gyroscope)	Amyloid deposits (brain) (implantable biosensor, wearable EEG)
Stroke	Gait, muscle tone, activity, impaired speech, memory (wearable EEG, accelerometer, gyroscope)	N/A
Diabetes	Visual impairment, sensory disturbance (wearable accelerometer, gyroscope)	Blood glucose level (implantable biosensor)
Rheumatoid Arthritis	Joint stiffness, reduced function, temperature (wearable accelerometer, gyroscope, thermistor)	Rheumatoid factor, inflammatory and auto-immune markers (implantable biosensor)
Renal Failure	Urine output (implantable bladder pressure/volume sensor)	Urea, creatine, potassium (implantable biosensor)
Vascular Disease (Peripheral Vascular and Aneurysms)	Peripheral perfusion, blood pressure, aneurysm sac pressure (wearable sensors)	Hemoglobin level (implantable biosensor)
Infectious Diseases	Body temperature (wearable thermistor)	Inflamatory markers, white cell count, pathogen metabolites (implantable biosensor)
Post-Operative Monitoring	Heart rate, blood pressure, ECG, oxygen saturation, temperature (wearable ECG sensor, thermistor and mechanoreceptor)	Hemoglobin, blood glucose, enzymes at the operative site (implantable biosensor)

**Table 4:** Requirements of wireless communication in implantable medical devices.

Network Parameter	Requirement of Implantable Medical Devices						
	Pace-Maker	Neural Stimulators	Drug-Delivery Systems	Retinal Implants	Cochlear Implants	Endoscopy Capsules	Active Prostheses
Throughput	up to 100 KBPS	up to 100 KBPS	up to 150 KBPS	up to 150 KBPS	up to 100 KBPS	up to 150 KBPS	up to 150 KBPS
Latency	up to 10 ms	up to 30 ms	up to 60 ms	up to 20 ms	up to 30 ms	up to 60 ms	up to 20 ms
Payload	40 KBPS	60 KBPS	30 KBPS	80 KBPS	60 KBPS	30 KBPS	80 KBPS
Duty cycling (MICS band)	0.1%	0.1%	0.25%	0.1%	0.1%	0.25%	0.1%

**Table 5:** Features of MAC protocols as suggested by IEEE 802.15.6.

Major Features of Efficient IBSN MAC	Acceptable Value for Implanted Medical Devices
Throughput	up to 200 KBPS for medical devices
	up to 4 Mbps for non-medical devices
Latency	up to 100 ms in life critical implants
	up to 2 s in monitoring medical devices
Bandwidth	300 KHz MICS band
	100 MHz in 2.4 GHz ISM band
	1.74 MHz in 433 MHz ISM band
Duty cycling	less than 0.01% in MICS band medical devices
	no restriction if Listen before talk is incorporated
Interference mitigation	CRC, frequency agility are recommended
	for safety purposes

**Table 6:** TDMA-based MAC protocols without WuR and their drawback for IBSN.

Name of the Protocol	Description	Special Feature	Potential Drawback
**LD-TDMA [30]**	Low duty-cycle TDMA	2.04 mW at 3 V DC using COTS transceiver. Power consumption is least compared to other protocols.	High latency in the event of a packet failure. Requires accurate synchronization
**HDMAC-TDMA [31]**	Heartbeat rhythm synchronized TDMA	Increased network lifetime by 15%–300% more than other similar BSN MAC	Suffers from severe single-point of failure problem. No accurate heart rhythm is measured all-over the body, hence use of network coordinator is necessary trading off with energy-efficiency and band-width efficiency.
**CF-MAC [32]**	Contention-free MAC protocol	Self-stabilizing and does not require a global time reference. The protocol will auto-stabilize for any network change	Cannot handle collision effectively, specially when a new node joins the network. Performance is severely affected on the event of change in network topology
**SSD-TDMA [33]**	Self-Stabilizing Deterministic TDMA	Energy efficient performance. Self stabilizing in case of dynamic data variations. Can support changes in network topology. Novel two layer approach for data-link creation.	Any slight violation in the assumptions made for the protocol will deviate the performance drastically. Cluster time synchronization is needed, directly proportioning to the performance. Some of the assumptions made cannot be met in real-world implementation
**HEH-MAC [34]**	Human Energy Harvesting MAC	Provides priority differentiation to the sensor nodes and flexibility to the network. Highly adaptive to environmental changes. Energy harvesting rates, network size and packet inter-arrival times are dynamically adapted within the protocol	Throughput, and other QoS parameters are not analyzed and presented. Suffers from global time synchronization issues, failing of which severly hinders the network parameters.
**PB-TDMA [35]**	Preamble-Based TDMA	Heterogeneous support for dynamic data. Can provide real-time guarantee. Very low energy consumption, yet less latency and high throughput is provided.	QoS depends on the preamble and time synchronization.
**BodyMAC [36]**	Energy efficient TDMA-based BSN MAC	BodyMAC uses flexible and efficient bandwidth allocation schemes with dynamic sleep mode. Supports dynamic applications in IBSN. Better performance in terms of the end- to-end packet delay and energy saving	No implementation is done. Results are based on software simulation. Highly accurate global synchronization is required.

**Table 7:** CSMA-based MAC protocols without WuR and their drawback for IBSN.

Name of the Protocol	Description	Special Feature	Potential Drawback
**CA-MAC [37]**	Context Adaptive MAC Protocol	CA-MAC is a novel approach of using a threshold value for deciding whether the packets are transmitted or not, based on the distance to the sink node. Energy efficient implementation for small scale dynamic network topology is achieved with CA-MAC. Latency is reduced by a novel adaptive algorithm based on the context of the packets.	Computational complexity is higher which is a threat to smaller resource constraint nodes and long-term network operation. Evaluation of the protocol is limited with theoretical data and ideal assumptions.
**PNP-MAC [38]**	Preemptive slot allocation and Non-Preemptive transmission MAC	Supports various types of traffics: continuous streaming, periodic data, time-critical emergency alarm, as well as non-eriodic data. Highly reliable QoS support. Novel combination of contention-free and contention access mechanisms.	Suffers from severe resource exhaustion. Energy consumption is not considered as a criteria for design. QoS will be traded off with energy efficiency and dynamic network topology.
**ULP-MAC [39]**	An Ultra-low-power Medium Access Control Protocol for Body Sensor Network	A cross-layer design strategy is adopted. Network coordinator and the sensors interact to achieve efficient power management. Variable super-frame structure is adapted. IBSN coordinator can make dynamic adjustment based on the feedback to achieve better performance in energy efficiency and latency.	Optimized for star topology. Suffers from hardware constraints such as memory and real-time guarantee. Simulation is carried out with ideal network conditions.
**B-MAC [40]**	Berkeley-MAC Versatile Low Power MAC protocol	BMAC renders properties of IBSN such as simple implementation on hardware, predictable performance parameters, and tolerance to network changes. Highly reliable data packet delivery of 98.5%	Very well suited for star topology networks. In case of change in network topology the protocol hinders performance [41]. Energy efficiency can only be expected when interfaced with different services resulting in cross-layer optimization.
**X-MAC [42]**	Short Preamble MAC Protocol for Duty-Cycled Wireless Sensor Networks	Low power communication is deployed by a strobed preamble approach that transmits a series of short preamble packets to the target receiver. Truncation the preamble by the target receiver saves energy at both the transmitter and receiver and introduce lower latency [41]. Near-optimal sleep and listen periods are demonstrated. X-MAC out-performs traditional Low-power listening techniques such B-MAC.	High latency in the event of a packet failure.
**DISSense [43]**	An adaptive, Ultra low-power MAC protocol	Cross-layer optimization issues are considered. Features such as data delivery ratio, latency, duty cycling and adaptability are better than other similar protocols. Can achieve good QoS in small scale networks.	Performance is traded off with energy consumption. No clear analysis of energy-efficiency is carried out. Designed for the purpose of large scale and coverage networks.
**MEB-MAC [44]**	Medical EmergencyBody (MEB) MAC	MEB-MAC focuses on the channel access delay reduction for medical emergency traffic with high reliability.	Implementation is done in real-world scenarios. However, no energy efficiency is concerned. It has adverse effect on new node insertion and mobility of network
**O-MAC [45]**	Ohio State University, Ohio-MAC	Increased energy efficiency by novel receiver scheduling methods such as Staggered On and Pseudo-randomized Staggered On. Theoretical analysis and practical implementation reveals that the protocol is 70% more energy efficient than B-MAC, S-MAC and T-MAC.	Qos is not considered, parameters such as latency and throughput are not evaluated.

**Table 8:** Hybrid MAC protocols without WuR and their drawback for IBSN.

Name of the Protocol	Description	Special Feature	Potential Drawback
**S-MAC [25]**	Sensor MAC	Good energy conserving properties with an ability to make trade-offs between energy and latency according to traffic conditions. The protocol has been implemented efficiently in hardware at real-world scenarios.	Scalability issues are not addressed. Network topology is considered constant with constant number of nodes.
**V-MAC [46]**	Virtual MAC	VMAC is embedded in Body QoS to make it radio-agnostic, so that it can control and schedule wireless resources without knowledge of the implementation details of the underlying MAC protocol. BodyQoS adopts an asymmetric architecture, in which most processing is done at the resourceful aggregator while less processing is done at the resource limited sensor nodes.	Energy efficiency is not considered at all. Evaluation of QoS parameters is given more importance than that of the energy concerns.
**DQBAN-MAC [47]**	Distributed Queuing Body Area Network MAC	High QoS support with limited protocol overhead. Less computational complexity and easy implementation. Novel integration of fuzzy rule scheduling along with TDMA-based approach renders a performance oriented cross-layer optimized MAC	Global time synchronization is a limiting factor. Power hungry due to extended operation of cross-layer optimization. Fuzzy logic will become a burden for the sensor nodes in case of dynamic data-load variations.
**R-MAC [48]**	Reservation Medium Access Control Protocol	Avoidance of overhearing, frequent commutation between sleep and wake up modes, and data collisions are good results of this novel approach. R-MAC protocol also adjusts the duration of the sleep and active periods according to the traffic load in order to avoid data collisions.	Not very energy efficient in low data rate application. Aimed at high data rate application in large scale networks
**UB-MAC [49]**	Urgency-based MAC Protocol	Critical nodes’ packet transmissions are prioritized over non critical nodes packet transmissions.	The proposed protocol is only evaluated mathematically. Network may fail for different network topology and number of nodes in a network is limited
**EEE-MAC [50]**	Energy Efficient Election-based MAC Protocol	Algorithm is good at preserving network topology and connectivity while introducing or reducing extra nodes. Smaller rate of deviation in energy consumption in higher data load conditions. Energy efficiency is good compared to S-MAC and B-MAC	The protocol is not analysed for QoS parameters. It is stated that QoS may hinder the energy efficiency for smaller networks
**FE-MAC [51]**	Forwarding Election-based MAC protocol	High network lifetime with energy efficiency and load balance. Routing capability of the network layer is also embedded in the protocol. Highly scalable and energy-efficient with more number of nodes	Resource utilization is exhaustive. Requires a relatively large memory and high computational power.
**QL-MAC [52]**	Q-learning-based MAC protocol	High data throughput is achieved. Support dynamic payload in variable network conditions. Computational complexity is minimal	Extremely high energy consumption. No mechanism to ensure QoS parameters in the protocol. No hardware implementation is done.
**RL-MAC [53]**	Reinforcement learning-based MAC protocol	QoS aware design. A total of 55% power savings is achieved in a star topology network.	Complex implementation of reinforcement learning algorithm to control the duty-cycle. Requires large resources to accumulate feedback from each transmission. Hardware implementation is not done

**Table 9:** Hybrid MAC protocols without WuR and their drawbacks for IBSN.

Name of the Protocol	Description	Special Feature	Potential Drawback
**Cooperative-MAC [54]**	Low duty-cycle TDMA	Suitable for highly mobile nodes. Novel combination of TDMA with FDMA deals with the interference and collision caused by the mobile cluster. The collisions brought by the mobile cluster are avoided through different frequencies used in WBAN	Complex hardware is required. Resource utilization is exhaustive rendering less power efficiency
**Hybrid-MAC [55]**	Hybrid (TDMA + FDMA) MAC Protocol	Reduced interference in the inter-cluster and intra-cluster communication using novel combination of FDMA and TDMA techniques. Achieves less energy consumption. Fulfills the bandwidth requirement of each node in the sensor network. Here after bandwidth division each node gets channel whose bandwidth is more than the requirement. Implementation is easy.	Less reliable, suffers from high packet drop for higher data load scenarios.
**Hy-MAC [56]**	Hybrid TDMA/FDMA MAC Protocol	A novel approach which schedules the network nodes in a way that eliminates collisions and provides small bounded end-to-end delay and high throughput. It takes advantage of multiple frequencies available in state-of-the-art sensor node hardware platforms such as MICAZ, TELOS and FireFly. Out-of-band synchronization is effective, rendering TDMA mechanism efficiently	Cannot be implemented in conservative radio band such as MICS where the number of channel available is highly limited. Not efficient in terms of energy
**HUA-MAC [57]**	Hybrid IBSN-Slot Access MAC Protocol	The special designed mini-slot method increases the contention efficiency. Contention-free data traffic scheme was adopted to guarantee the QoS. Allocation of slots is adaptive to the traffic load. Increased scalability and robustness for a BAN.	Suffers from severe limitations from state-of-the-art hardware. Real-world implementation was carried out with ideal assumptions of network parameters. Energy efficiency is lagging
**YNU-MAC [58]**	YNU Japan, Ultra-WideBand MAC proposal	Protocol considers SAR or thermal influence to human body by switching cluster mechanism. Positioning or localization of BAN nodes is highly possible	Different supplementary technologies yet to be analyzed. Implementation is not possible with COTS hardware
**FM-UWB MAC [59]**	CSEM Switzerland, Frequency Modulation—Ultra WideBand MAC proposal	Low energy at the transmitter and also saves energy at the destination node as it does not have to listen to a complete wake-up preamble. Suffers less from overhearing. Reduced channel usage and thereby collisions. Improved reliability and reduced latency	Extreme requirement for hardware compared to other mechanisms. No optimal physical layer design is proposed

**Table 10:** MAC protocols with WuR and their drawbacks for IBSN.

Name of the Protocol	Description	Special Feature	Potential Drawback
**NICT-MAC [60]**	NICT Japan, Proposal for MAC using WuR	Provides QoS guarantee for the most important life-critical message and majority real-time traffic. Can be used in different physical layers UWB, MICS, WMTS, HBC. A dynamic network size from greater than six nodes to less than 100 nodes per network can be achieved.	Power consumption higher due to high performance. ALOHA is used instead of CSMA/CA which may result in abrupt performance deviation in large network size.
**IMEC-MAC [61]**	IMEC Narrow band MAC proposal	Improved QoS addressing throughput, access latency, priority. High scalability is realized. Star, cluster-tree and the peer-to-peer, are supported.	The design is prone to collison and Low resource efficiency. Energy consumption is very high compared to other similar protocols.
**Miller-MAC [62]**	A MAC Protocol to Reduce Sensor Network Energy Consumption Using a Wakeup Radio	Supports multiple hop and multiple flow scenarios, outperforming similar protocols in terms of energy and latency.	Additional hardware is required to allow senders to force receivers to wake-up when a specified number of packets are buffered. Extra hardware consumed extra energy, which is not analyzed in the design.
**RTWAC-MAC [63]**	Radio Triggered wake-up with Addressing Capabilities MAC	Reduces idle listening and also suppress unnecessary radio wake-ups due to the addressing information included in the wake-up signal. Very less power consumption and low latency than other MAC protocols (SMAC TMAC and BMAC)	No explicit analysis of QoS with respect to the energy consumption. Further work is needed to integrate with different MAC protocols for data communication using main radio.
**PE-MAC [64]**	Power efficient MAC using WuR	Uses TDMA-based MAC with wakeup radio that can save a more than 50% of energy used in CSMA/CA while still having a low delay in data transfer	Higher network size is not considered, rather only two nodes were used to evaluate the protocol. QoS and other network parameters such as interference, delay are ideally assumed in the evaluation.
**ULPA-MAC [65]**	Ultra Low Power Asynchronous MAC Protocol using WuR	This approach can improve up to 82% QoS and 53% energy saving when considering with TICER [66] protocol for wireless communications.	Suffers from severe data and wake-up beacon collisions in a high traffic network, which reduces the average data received rate by 5.39%.
**WuR MAC [66]**	Wake-up radio MAC	By eliminating polling for detecting channel activity, this method provides more energy-efficient solutions than B-MAC and S-MAC. Very low per-hop latency and average power consumption	Mathematical analysis is done rather than real world implementation. Real-world difficulties are bypassed with assumptions.
**T-MAC [67]**	Adaptive Energy-Efficient MAC Protocol	Handles load variations in time and location by adaptive duty-cycle in a novel way. Reduces the amount of energy wasted on idle listening	Throughput is traded off with energy efficiency. Experimented in static and non-mobile networks
**TBCD-TDM [68]**	Time-Based Coded Data-Time Division Multiplexing	A 280 times higher throughput than ZigBee protocol. Simple modulation techniques, requires very less hardware complexity	Ideal case of only one single transmitted data bit per round is used. No real-world implementation is discussed such as effect of environment noise effect.

**Table 11:** Comparision of MAC protocols. Legends: ‘+’ = Positive; ‘−’ = Negative; ‘*O*’ = Neutral.

MAC Protocol	Features						
	Energy-Efficiency	Reliability	Overhead	Effective-Throughput	Low-Latency	Hardware Complexity	Access Mechanism
CF-MAC [32] Y-2005	+	+	+	−	+	+	TDMA
SSD-TDMA [33] Y-2005	+	−	*O*	−	+	+	TDMA
DQBAN MAC [47] Y-2009	−	−	+	+	−	*O*	TDMA
HEH-MAC [34] Y-2007	−	*O*	−	*O*	+	+	TDMA
BodyMAC [36] Y-2009	+	+	−	+	*O*	−	TDMA
UB-MAC [49] Y-2010	−	+	−	+	−	+	TDMA + CSMA
X-MAC [42] Y-2006	*O*	−	+	−	+	+	CSMA
V-MAC [46] Y-2008	+	−	+	−	+	*O*	TDMA + CSMA
R-MAC [48] Y-2007	+	+	−	−	*O*	+	TDMA + CSMA
PNP-MAC [38] Y-2010	*O*	−	+	+	−	+	TDMA + CSMA
O-MAC [45] Y-2006	+	−	−	−	+	−	TDMA + CSMA
MEB-MAC [44] Y-2012	+	+	+	−	+	−	TDMA + CSMA
EEE-MAC [50] Y-2013	−	+	*O*	−	+	−	TDMA + CSMA
FE-MAC [51] Y-2007	+	−	+	+	−	−	TDMA + CSMA
P-MAC [72] Y-2013	+	−	+	+	+	*O*	TDMA + CSMA
CA-MAC [37] Y-2009	−	+	−	*O*	+	−	TDMA + CSMA
ULP-MAC [39] Y-2005	+	+	−	+	*O*	+	TDMA + CSMA
BMAC [40] Y-2007	*O*	+	+	−	+	−	CSMA
BSN-MAC [57] Y-2010	+	−	*O*	+	+	−	TDMA + CSMA
ULPD-MAC [73] Y-2008	−	+	+	−	*O*	*O*	TDMA + CSMA
DISSense [43] Y-2007	+	*O*	+	−	+	+	TDMA + CSMA
S-MAC [25] Y-2002	−	+	+	−	+	−	TDMA + CSMA
Cooperative—MAC [74] Y-2008	+	+	−	+	−	−	TDMA + FDMA
Hybrid-MAC [55] Y-2014	+	−	+	+	−	+	TDMA + FDMA
HyMAC [56] Y-2012	+	*O*	+	−	*O*	+	TDMA + FDMA
HUA-MAC [57] Y-2010	+	+	−	−	−	*O*	Hybrid ALOHA
YNU-MAC [58] Y-2009	−	+	+	+	+	−	CSMA + UWB
FM-UWB MAC [59] Y-2009	−	−	−	*O*	−	+	CSMA + UWB
RL-MAC [53] Y-2006	+	−	−	−	−	+	CSMA + Adaptive learning
QL-MAC [52] Y-2013	+	−	+	*O*	−	+	CSMA + Adaptive learning
NICT-MAC [60] Y-2014	+	+	−	+	*O*	+	Slotted ALOHA + WuR
IMEC-MAC [61] Y-2009	−	+	−	−	−	*O*	ALOHA + TDMA + WuR
Miller-MAC [62] Y-2005	+	*O*	−	−	−	+	TDMA + CSMA + WuR
RTWAC [63] Y-2009	+	+	+	+	+	+	TDMA + CSMA + WuR
PE-MAC [64] Y-2011	*O*	−	−	+	−	−	TDMA + CSMA + WuR
ULPA-MAC [65] Y-2013	+	−	+	*O*	−	+	CSMA + WuR
WuR MAC [66] Y-2004	+	−	*O*	−	+	*O*	CSMA + WuR
T-MAC [67] Y-2003	−	−	+	+	−	+	CSMA + WuR
TBCD-TDM [68] Y-2009	+	+	−	+	−	+	TDMA + WuR

## Abbreviations

The following abbreviations are used in this manuscript:

MAC	Medium Access Control
QoS	Quality of Service
DBS	Deep Brain Stimulator
WHO	World Health Organization
BSN	Body Sensor Network
BAN	Body Area Network
PAN	Personal Area Network
IBSN	Implantable Body Sensor Networks
WSN	Wireless Sensor Network
UWB	Ultra Wide Band
MICS	Medical Implant Communication Service
CSMA	Carrier Sense Multiple Access
TDMA	Time Division Multiple Access
FDMA	Frequency Division Multiple Access
WuR	Wake-up Radio
